# Loss of human life by suicide – lived experiences of suicide survivors and professionals

**DOI:** 10.1080/17482631.2026.2713239

**Published:** 2026-08-03

**Authors:** Christina Nilsson, Anders Bremer, Karin Blomberg, Elisabeth Bergdahl

**Affiliations:** a Faculty of Medicine and Health, School of Health Sciences, Örebro University, Örebro, Sweden; b Division of Emergency Medical Services and University Health Care Research Center, Örebro University Hospital, Örebro, Sweden; c Faculty of Health and Life Sciences, Department of Health and Caring Sciences, Linnaeus University, Växjö, Sweden

**Keywords:** Existential suffering, lived experience, participatory action research, phenomenology, professionals, suicide survivors

## Abstract

**Purpose:**

This study aimed to describe the phenomenon of losing human life by suicide, based on the lived experiences of suicide survivors and professionals.

**Methods:**

Phenomenological participatory action research was conducted, comprising 50 group sessions with 51 participants including suicide survivors, registered nurses, response police officers, and general practitioners.

**Results:**

The essence of losing human life through suicide is characterized by profound emotional vulnerability when confronted with an existential tragedy. This means a powerlessness and an abrupt shift in existence, awakening the instinct to escape and the responsibility to act. Being the messenger of death is a heavy burden, and a sense of abandonment arises when survivors are left alone. Simply being together as human beings awakens a sense of vulnerability and a strong impulse to uphold human dignity.

**Conclusions:**

These findings underscore that everyone involved is vulnerable to existential challenges following suicide and provide insight into the fragility of humanity. Professionals need awareness of their own vulnerability and moral courage to encounter survivors as fellow human beings. Support for survivors should acknowledge that grief and existential suffering do not follow fixed timelines. The methodological combination of phenomenology and participatory action research proved fruitful, generating rich insights and offering a promising approach for future studies.

## Introduction

Every year, more than 720,000 people worldwide die by suicide (World Health Organisation, 2025). Each suicide is estimated to affect roughly 135 people on average, although the number of people whose lives are permanently altered by traumatic experiences and losses remains unknown (Cerel et al., 2019). In this article, a person who has lost a family member to suicide is referred to as a *suicide survivor*.

Most suicides occur in prehospital settings, where ambulance personnel may withhold resuscitation or terminate resuscitation efforts on the scene in accordance with the guidelines (Mentzelopoulos et al., 2021). However, termination or withholding of resuscitation does not automatically mean that the bereaved family member is considered as a patient. In the event of death, ambulance personnel are responsible for pronouncing death (Risson et al., 2023), whereas a medical practitioner is usually responsible for issuing the death certificate (World Health Organisation, 2023). When delivering a death notification, two perspectives are critical: that of the notifier and that of the recipient (De Leo et al., 2015). The professionals most commonly tasked with the delivery of death notifications are doctors, police officers, and registered nurses (De Leo et al., 2020).

Previous research has highlighted the emotional and professional challenges associated with such encounters. In an interview study, police officers described feeling helpless and frustrated when confronted with suicide, as they were unable to make sense of the event or provide help (Koch, 2010). According to doctors, registered nurses, and police officers, the delivery of a death notification following a violent or unexpected death is particularly challenging, often evoking feelings of inadequacy and emotional strain (De Leo et al., 2022). Suicide is consistently ranked among the most emotionally demanding causes of death for professionals such as police officers, victim advocates, chaplains, social workers, and psychologists to handle (Stewart et al., 2000). For police officers, delivering a death notification is perceived as a significant burden (Hofmann et al., 2022), whereas general practitioners (GPs) and emergency medical service providers often report discomfort when notifying and interacting with bereaved families (Foggin et al., 2016; Smith-Cumberland & Feldman, 2005; Tataris et al., 2017). GPs have described feeling unprepared to meet parents bereaved by suicide (Foggin et al., 2016). Among police officers, two of the most frequently reported critical incidents are the delivery of death notifications and responses to suicides involving adults (Møller et al., 2023). Despite these challenges, training and procedures for delivering death notifications remain insufficient (Campos et al., 2021; De Leo et al., 2022; Foggin et al., 2016; Risson et al., 2023; Stewart et al., 2000; Tataris et al., 2017). Unpreparedness for delivering death notifications and encountering bereaved family members may lead to anxiety, avoidance, and emotional distancing among ambulance personnel. Mentzelopoulos et al. (2021) emphasises the need for further research to identify how ambulance personnel can best be prepared and supported in managing patient death within the prehospital context.

For suicide survivors, the aftermath of suicide often involves profound and enduring suffering. Typical reactions include the feeling of being left with unanswered questions about why people end their lives (Pompili et al., 2013). From the perspective of those who have lost a family member to suicide, the experience is often marked by profound existential loneliness and suffering, a form of distress that threatens one’s sense of meaning, dignity, and identity. The grieving process typically involves prolonged and energy-consuming efforts to make sense of the loss while simultaneously navigating new and often overwhelming burdens in a life that has been radically altered (Nilsson et al., 2022). A systematic review found that grief following suicide differed significantly from grief after other types of death, particularly regarding feelings of rejection, shame, stigma, blaming, and concealing the cause of death (Sveen & Walby, 2008). A family affected by suicide has a higher risk of being affected by suicide again (Niederkrotenthaler et al., 2012; Qin et al., 2002; Runeson & Asberg, 2003; Tidemalm et al., 2011). Professionals’ behaviour during death notification can profoundly influence outcomes (Hofmann et al., 2023; Stewart et al., 2000). The actions taken by notifiers may either intensify trauma and loss, or mitigate their impact (De Leo et al., 2022).

Given this background, there is a need to advance knowledge of the phenomenon of suicide loss as it is experienced by those directly affected. Although research has examined the experiences of suicide survivors and professionals separately, little is known about how these experiences converge and diverge during the early period following a suicide and about the consequences that unfold in its aftermath. Exploring these perspectives together may provide a richer and more nuanced understanding of the phenomenon. Therefore, this study aimed to describe the phenomenon of losing human life by suicide, based on the lived experiences of suicide survivors and professionals.

## Research design

A phenomenological method was used, based on reflective lifeworld research (RLR) according to Dahlberg et al. (2008) in combination with participatory action research (PAR) based on the work of McNiff and Whitehead (2011). RLR constituted the overarching theoretical and methodological foundation of the study (Dahlberg et al., 2008). The methodological principles of RLR—openness, curiosity, and bridling—guided the researchers throughout all stages of the research process to identify and describe essential meanings of the phenomenon, grounded in lived experiences.

## Method

### Recruitment of participants

The participants in this study were suicide survivors and professionals who had encountered suicide survivors in the initial phase following suicide. The inclusion criteria for suicide survivors were being a bereaved family member of a person who died by suicide outside the hospital and being over 18 years of age. Being a family member was defined as being part of a self-defined group of two or more individuals functioning in such a way that they perceived themselves as a family (Whall, 1986).

Suicide survivors were recruited through advertisements in two daily newspapers on four occasions and through social media (Facebook). Potential participants were invited to send an email to the first author (CN) to show their interest in participation, and an information letter about the study was sent to them in response. Of the 53 survivors who received the information letter, five were excluded as they did not meet the inclusion criteria, 15 declined to participate, and seven did not contact the researchers after receiving the information letter. The remaining 26 survivors participated in the study (Table I); 22 were women, the mean age was 56 (range: 27–88) years, and suicide had taken place a mean of 18 (range: 0–57) years previously. There was variation in their family relationships with the person who had died (daughter, sister, mother, father, son, brother, wife, and partner), and the type of suicide also varied. Most of the deceased were men.

**Table I.** Participants in the phenomenological participatory action research process, and the development of the process in the group sessions.

	First sessions	Second sessions	Third sessions	Fourth sessions
**Number of participants**				
Suicide survivors	25	20	13	17
Ambulance nurses	14	13	13	12
Response police officers	9	6	5	6
General practitioners	2	1	–	1
**Total**	**50**	**40**	**31**	**36**
**Content of the group session**				
Narrative	x	x		
Story/dialogue method	x			
Reflective lifeworld research	x	x	x	x
Reflect	x	x	x	x
Observe	x	x	x	x
Act		x	x	x
Evaluate		x	x	x
Modify		x	x	x
Move in new directions			x	x

The inclusion criteria for professionals were (1) being ambulance personnel, a response police officer, or a GP; and (2) having encountered suicide survivors outside the hospital in their professional practice. This study was conducted in a medium-sized region in Sweden. Approval for data collection was granted by the police authority and the managers of the health care organisation.

All ambulance personnel, response police officers, and GPs received information at staff meetings, and a video describing the project was sent to their work email together with an information letter. Potential participants were asked to contact the first author (CN) if they were interested. A total of 25 professionals participated in the study; 14 were men, the mean age was 37 (range: 27–56) years, and their mean length of time in their profession was nine (range: 1–20) years. Fourteen were registered nurses (henceforth referred to as “nurses”), nine were response police officers, and two were GPs (Table I). Thirteen nurses had received 1-year specialist training in ambulance care to become a prehospital emergency nurse. For security reasons, response police officers who were interested in participating were asked to contact the chief of the police. The chief sent contact information for these officers to the first author (CN), along with permission from the researchers to contact them, and a date for the first session was then set.

### Data gathering

The participants (*n* = 50) in the phenomenological PAR process were suicide survivors, nurses, response police officers, and GPs, who participated in a total of 50 group sessions. All groups were homogeneous, in the sense that survivors and professionals were not mixed, and participants did not switch between groups. The distribution of the groups and participants is shown in Table I.

Additionally, three individual face-to-face interviews were conducted (Table I). One suicide survivor participated only in an interview; hence, a total of 51 persons participated in the study. The total time for all sessions and interviews was 60 hours and 37 minutes (Table II). Data were collected between June 2022 and April 2024 from 13 groups, 11 of which met on four occasions and two of which (the GPs and one of the three groups of response police officers) met on three occasions.

**Table II.** Number and duration of sessions and face-to-face interviews for each type of participant.

Group composition	Groups (*n*)	Total sessions (*n*)	Face-to-face interviews (*n*)	Total duration of sessions
Suicide survivors	5	20	2	30 h 32 min
Ambulance nurses	4	16	1	17 h 28 min
Response police officers	3	11	–	10 h 54 min
General practitioners	1	3	–	1 h 43 min
**Total**	**13**	**50**	**3**	**60 h 37 min**

### Phenomenological participatory action research process

**Figure 1.**
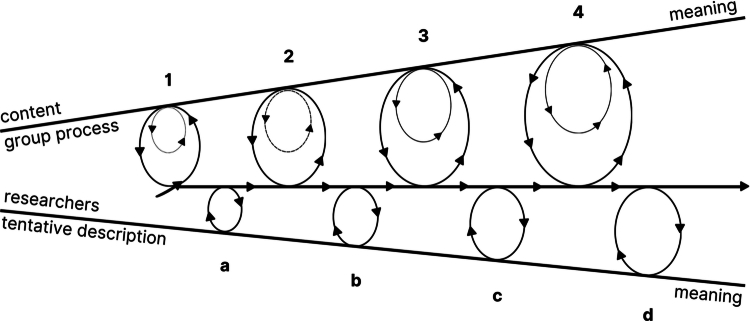
Illustration of the iterative approach to meaning making through four cycles of group process (1–4) and researcher analysis (a–d).

The phenomenological PAR process is cyclical rather than linear, and each group session likewise followed a reflective, cyclical process (Figure 1). Throughout the process, the action-reflection cycle was used, consisting of the phases *reflect-observe-act-evaluate-modify-move in new directions* (McNiff & Whitehead, 2011) (Table I).

The researchers maintained a high level of trust in the participants’ abilities throughout the process and PAR made it possible to transfer responsibility to the groups in each session. The first author allowed space for the group and stayed silent when the group engaged independently. This approach gave participants both the mandate and the motivation to take responsibility. It also enabled their experiences to be expressed and explored in their own words. This approach is in line with RLR, as it signals curiosity about what others have to say and openness to the new and unfamiliar. Likewise, the approach is consistent with PAR, with an evident bottom-up perspective and transfer of power to participants in the group sessions. In Figure 1, cycles 1–4 describe the group sessions and cycles a–d describe the researchers’ analysis of the data collected after each session. Table III provides an overview of all groups in the process and the analysis.

**Table III.** An overview of the groups, the process and analysis.

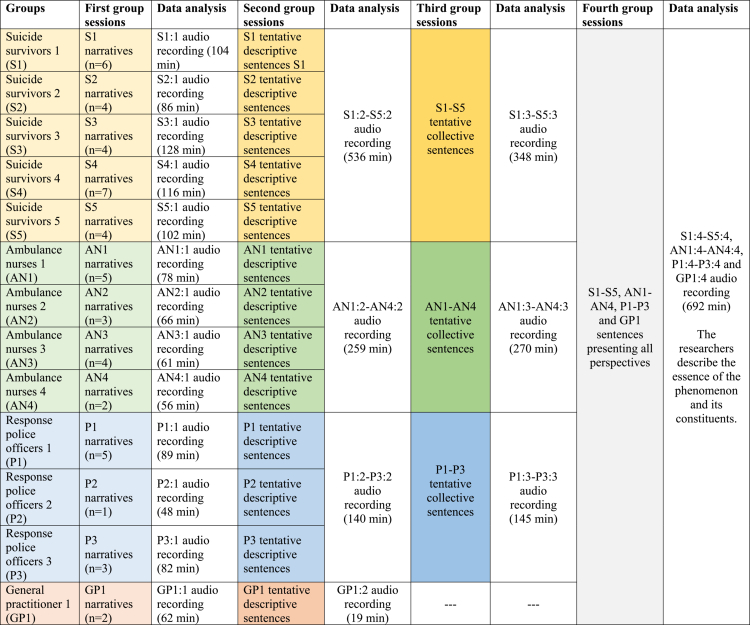

### The first group sessions: the participants’ narratives

Initially, everyone introduced themselves briefly, and the group made a joint decision on confidentiality. A broad opening question was asked in line with phenomenology (Dahlberg et al., 2008) to encourage the participants to share their stories. The survivors were asked, “*Could one of you tell us your story based on your experiences?*” The professionals were asked: “*Based on your experiences, can anyone tell me about a suicide situation with suicide survivors?”* In each group, participants shared their experiences to the best of their ability (Figure 1). Probing questions were employed to deepen participants’ descriptions and clarify meanings, for example: *“Can you describe a little more about ...?”* and *“Can you tell me more about what you mean by ...?”* They described experiences related to the course of events, and their feelings and thoughts. Their narratives were rich, touching, and deep. Participants recognised themselves in the narratives of others. They listened actively and intensively to each other, confirmed each other, and reflected spontaneously on what they heard in relation to their own experiences. Early on, there was the creation of togetherness, compassion, and support between the members of each group. A permissive atmosphere existed in all groups, allowing breaks and support when a participant became emotional. On these occasions, the group waited for the person to compose themselves, and when they felt ready, the narrative continued.

At the end of the session, the story/dialogue method inspired by Labonte et al., (1999) was used to created insights. An insight is an understanding of the moment that is worth sharing with others (Labonte & Feather, 1996). Participant first wrote down their insight individually on a piece of paper to help them initiate and deepen their self-reflection. Most participants chose to share their insights to the group, and reflections were met with clear affirmation from other participants.

### The researchers’ first analysis

The first author listened to all audio recordings several times (Table III) and applied live coding (Parameswaran et al., 2020). NVivo (QSR International, 2018) was used to manage the extensive dataset. The analysis focused on the content, with the researchers’ descriptions remaining closely aligned with the participants’ accounts to preserve the group’s way of articulating their experiences. Tentative descriptive sentences were formulated for each group. If there were any parts that were not entirely clear or when different parts pointed in different directions, questions were formulated for each group.

### The second group sessions: tentative descriptive sentences

The session began with a round of reflection, during which participants were invited to share any new thoughts that had emerged since the previous session or anything else they wished to add. The first author made it clear that the sentences presented were tentative attempts to understand the content and not necessarily entirely accurate. The participants were told that they should feel free to change a sentence if they did not recognise themselves in relation to what they had experienced, and to write completely new sentences on pieces of paper if anything was missing. One tentative descriptive sentence at time was presented to the group by the first author, who read it aloud and placed it on the table for the group to observe. Next, the group began to reflect on what they recognised and what they did not, through reasoning back and forth between themselves and each other. Sometimes the participants argued for another participant’s voice, as they knew each other’s stories. They worked by evaluating and correcting the text, adjusting or modifying it as needed, and in some cases providing entirely new sentences when details or nuances were missing. They reflected together again and thus deepened their narratives in terms of similarities and differences. The first author was asked open-ended questions when needed to get the group to continue forward and develop their narrative, and for clarification. The participants also asked each other in-depth questions based on their own experiences. The reflections became refined and transparent, which can be seen as double loop reflections (Torbert & Taylor, 2008). This is a form of reflection that goes deep and can increase insights into complex events. When all the sentences had been presented and the participants were asked to group the sentences and then describe their groupings.

### The researchers’ second analysis

The first author listened to all audio recordings several times (Table III). The sentences were reviewed and revised by the researchers to incorporate participants’ corrections and additions. Through this process, a focus on shared understanding began to emerge. Based on tentative descriptive sentences, tentative collective sentences for the survivor and professional groups were developed. Where aspects were unclear or where different parts appeared inconsistent, questions were formulated for the group.

### The third group sessions: tentative collective sentences

The session began also with a round of reflection, during which participants were invited to share any new thoughts that had emerged since the previous session or anything else they wished to add. Each group expressed curiosity about the perspectives of the other groups on the same issues. The first author reiterated that the presented sentences were tentative attempts to understand meaning and were not necessarily entirely accurate. The researchers read one tentative collective sentence at a time and then put it down in front of the group for them to observe. The survivors transitioned from considering their personal narratives to identifying common elements within the stories of all participants. This set the path for reasoning to move towards a more common understanding of the nuances of the phenomenon. The professionals moved beyond simply describing a job task to more personal thoughts and perspectives by describing what made these events challenging. They highlighted complex emotional challenges, experienced both internally and in relation to their encounters with survivors. The process involved personal and shared reflection, mutual affirmation, and iterative discussion of their narratives.

Participants independently reached higher levels of abstraction in the reflection process, with groups naturally using more complex terminology in a shared understanding. They recognised themselves in the presented sentences but sometimes felt the need to clarify and deepen their narratives. For example, when someone in the group did not think a sentence matched what they had experienced, others in the group helped find more nuanced words that better illuminated the experience. The discussions were valuable, and the group actively worked on the new broader sentences, which gave them new insights as well as giving the sentences more depth and gradually leading them towards meaning. This cycle also became a reflective double loop. When all collective sentences had been presented and the participants had given their comments and made changes, they were asked to group the sentences and describe their groupings to increase their understanding and thereby move in a new direction.

### The researchers’ third analysis

The first author listened to all the audio recordings several times (Table III). The sentences were revised by the researchers to incorporate participants’ corrections and additions, thereby conveying their meanings.

### The fourth group sessions: sentences presenting all perspectives

This session also marked the beginning of a period of reflection, during which participants were invited to share any new thoughts that had emerged since the previous session or any additional comments. There was a feeling of excitement and curiosity in the room about being able to share experiences and insights from all perspectives. Participants received one piece of paper at a time, each containing collective sentences from one of the four perspectives. Once again, the participants actively engaged and participated. All participants reflected on, commented on, evaluated, understood more about, and were sometimes surprised by other groups’ descriptions. This further deepened the shared experiences. When sentences formulating all perspectives had been presented, and the participants had given their comments, they were asked to arrange these sentences in front of them and reflect on the whole. New insights were then formulated by the survivors and professionals, showing individual lessons learned which might influence future actions and move in new directions.

### Individual face-to-face interviews

The three face-to-face interviews were conducted for different reasons. One suicide survivor wished to share their experiences individually. Another interview involved a suicide survivor who had been very quiet during the group session, and who was therefore offered an individual interview to express their experiences more fully. Finally, one nurse was the only person able to attend the scheduled session time due to the work schedules of the other group members; this nurse still wished to be interviewed, and the researcher therefore accommodated this request. The question that was asked in the first session was also the opening question in the interview, i.e., to survivors “*Could one of you tell us your story based on your experiences?*” and to professionals “*Based on your experiences, can anyone tell me about a suicide situation with suicide survivors?”* Probing questions were employed to deepen participants’ descriptions and clarify meanings.

### The researchers’ analysis on the face-to-face interviews

The first author listened to all audio recordings several times. With a focus on the phenomenon, the researchers developed descriptions that captured essential, nuanced, and varied meanings. The analysis of the interviews was then integrated with the meanings of the groups, adding further nuance.

### The researchers’ final analysis

Once all sessions and individual interviews were conducted, the researchers continued the data analysis, as the meanings needed to be clarified, deepened, and abstracted even more so that the results could be understood by others outside the groups and provide scientific knowledge. Based on the participants’ stories and the work undertaken during the group sessions, the researchers extracted varying meanings of the phenomenon building its essence and constituents, following the RLR approach (Dahlberg et al., 2008), avoiding comparative analysis. The constituents reveal both the variations and the shared meanings, supported by direct quotations from the participants’ narratives.

## Ethical considerations

The researchers were fully aware that the participants could regard the topic as being sensitive. Therefore, all suicide survivors received the phone number of a counsellor to use if they needed further support before or after the sessions. For the professional groups, the relevant occupational healthcare services were informed about the study. All participants were informed in writing and orally that participation was voluntary, and that they had the right at any time to refrain from participating without having to give reasons. Written informed consent was obtained from all the participants. Before initiating data collection, participants consented to treat all information as confidential. At the sessions, the researchers were attentive to the group and adapted to their needs. Each session ended by asking everyone in the group how they had experienced it. The group members were then asked if they wanted to meet again, and if so, another session was booked. Study approved by the Swedish Ethical Review Authority (No. 2022-00802-01 and No. 2022-02535-02).

## Results

### Essence

The loss of human life through suicide is permeated by the emotional nakedness of being faced with existential tragedy resulting in a deep embodied experience. The deliberate ending of life brings with it an unavoidable awareness of a profound and abrupt shift in existence—a shift often accompanied by suffering and a deep sense of vulnerability in simply being together as human beings. Powerlessness prevails in a situation where a person’s life cannot be saved, and where inadequacy and a brutal reality awaken the instinct to escape, but also to actively take responsibility. Being the messenger of death evokes a profound emotional burden, often accompanied by a sense of abandonment when survivors, particularly children, are left to face their grief alone. A strong ethical impulse emerges to uphold human dignity by shifting the focus away from the manner of death and instead affirming the intrinsic value of the person and the depth of their loss. The tragedy of suicide is unavoidable for those who face tangible powerlessness, responsibility in solitude, embodied suffering that emerged as a memory while expressing a strong drive to maintain human dignity.

### Tangible powerlessness

Suicide confronts the most profound and painful dimensions of human existence and represents a deliberate act that leads to the end of one’s life. When a person’s life cannot be saved, tangible powerlessness arises for everyone affected with feelings of incomprehensibility and inadequacy. There is a definitive change in life that places people in vulnerable situations that are not self-chosen.

Suicide is an event that seems incomprehensible, with feelings of time standing still and unreality devolving into merely trying to take in what has happened. Not being allowed to share the process of death is difficult and creates feelings of exclusion. One survivor expressed her indignation over this powerlessness:

*It affects me! It affects me! In this type of death, one is not allowed to participate.*

Suicide often occurs suddenly and unpredictably, leaving those affected unprepared, although some individuals may repeatedly encounter it. The responsibility of delivering the death notification typically falls on the legally designated person, which can mean that the message is not conveyed to the individual who is emotionally closest to the deceased, but rather to the person recognised as the legal next of kin. The abrupt transition from life to death creates a situation that feels like a fait accompli—the reality that the person’s life cannot be saved is immediate and irreversible. This moment is marked by a profound sense of powerlessness, followed by emotional tension surrounding the act of announcing a death. A nurse described his lack of power:

*Cardiac arrests often involve elderly and sick people, and there’s a naturalness in these deaths. However, that isn’t the case here. It’s wrong at every level: emotional, medical, and social. Wrong timing. This person should not have died.*

Loss by suicide is a unique and emotionally charged event, and there is only one opportunity to do the right thing, knowing that the moment when the death notice is given will be a lifelong memory. The death notification is provided without reservation, using the unambiguous word “death”. Because it is impossible to give a positive message, the negative and tragic messages are conveyed in the best way possible. Formal and practical tasks take up a large part of the time, and sometimes clarifying questions must be asked to rule out crime. However, even if professionals want to answer survivors’ questions, they can be limited by laws that dictate what can be said and whether the body can be shown. Despite feelings of leaving survivors to themselves, the harsh reality and situation make this impossible to avoid. The responsibility for dealing with survivors and being a fellow human sometimes becomes an extra task that is difficult to manage, leaving a nagging and profound sense of inadequacy. A response police officer expressed inadequacy bordering on powerlessness:

*In terms of work, you want to solve everything practical regarding the body and other things, but your heart goes to the family members who are outside [the house]. They are the ones you need to spend time on.*

Having tried for a long time but failed to get help for the deceased person’s psychiatric illness felt contradictory when compared with health care resources spent on life-saving interventions even in situations where saving a life is not possible. One bereaved person described his powerlessness in terms of missing information that deprived him of the opportunity to help or understand the deceased person’s suffering in time:

*The hardest part was that I didn’t know about it before. Maybe you [I] could have done something, been there, and helped. They [the medical staff] knew everything; I knew nothing. Actually, I thought that this was somewhat difficult.*

A vacuum of uncertainty and confusion can arise when several days pass before a death can be confirmed and a death notification is issued. Frustration intensifies when the most basic questions remain unanswered. Being denied access to the deceased person’s body means being deprived of the possibility to understand, to be convinced that the person has died, and to avoid fantasies. Children end up in a particularly exposed situation; their lives change, and they are totally dependent on the ability of other family members to support them. There are external expectations and demands that life should continue as usual, without any real understanding that suicide is a life-changing event that involves more than loss and grief. Tangible powerlessness can affect those closest to the deceased in a naked and brutal way, and this powerlessness grips deeply when it becomes clear that a close person’s active action against themself has forever changed others’ lives. One survivor described the total and tangible powerlessness:

*I got a phone call: “Now I’ve made up my mind.”*

*I understood immediately and said: “Please, please come home!”*

*“No.”*

*He turned off the phone.*

### Responsibility in solitude

Responsibility in solitude refers to the inner conflict between actively taking responsibility and bearing the weight of loss, while simultaneously grappling with uncertainty about one’s capacity to do so and managing a sense of vulnerability in simply being together as human beings. When bereaved people are expected to support others in grief, this can evoke a profound sense of abandonment by those around them and by society at large. In the absence of someone willing to shoulder this responsibility, children are often left to navigate their grief on their own.

A sense of loneliness in relation to responsibility is experienced by those tasked with delivering death notifications to survivors. The uncertainty of what and who awaits at the scene of the suicide, combined with the fear that nervousness may take over and lead to a loss of control, creates doubt about one’s ability to offer meaningful support. At such moments, humanity hangs by a fragile thread, and the transition from care governed by strict protocols concerning the deceased’s body to the more ambiguous task of supporting survivors can be challenging. Formal responsibilities and legal procedures carry significant weight, in stark contrast to the unregulated and emotionally demanding task of providing support to the bereaved. For the latter task to be prioritised, an active choice must be made, often requiring those with the most experience to assume greater responsibility. Insecurity arises when the boundaries of one’s expected professional role become blurred:

*It’s the uncertainty. I’m afraid of making things worse. This limits my ability to provide support to survivors.*

Humanity in the professional role is valued in relation to its consequences for one’s own wellbeing, and discomfort is weighed against the value of engaging as a fellow human being. A calm and level-headed colleague can be crucial for commitment and courage to remain in an existential void. Collegial support is crucial, and if it is lacking, the ability to self-reflect is important. Despite emotional strain, professionals often feel responsible not only for their own wellbeing but also for supporting others when the situation demands it. Through reflection, a sense of gratitude may arise for life itself and for what exists beyond the boundaries of professional practice. Survivors who receive the death notification are often left with the responsibility of informing other family members, thereby becoming accountable for both their own and others’ emotional wellbeing over time. This creates a form of double suffering, in which the bereaved must simultaneously endure their own grief while providing support within and beyond the family. When parents or other adults close to children are overwhelmed by their own grief and unable to support their children in mourning or everyday life, the burden of responsibility may fall on the children themselves. In such cases, the absence of adult support leaves children to navigate their grief alone, highlighting the painful failure of the adult world to take responsibility.

*There was nobody [who could provide support]. It was just going back to school and not being able to talk to anyone and not knowing where to turn. And no one seems to ask if you need any kind of support/…/but leaves us with our mother, who felt so terribly unwell. It was left for us, children, me and my siblings, to figure it all out ourselves.*

Abandonment is experienced by those who have not received support, leaving a vacuum of unfulfilled needs that must be seen and affirmed. Family members find it difficult to support each other, and lack support from someone outside the family who can relieve them and be available over time. Shouldering responsibility becomes even clearer when surrounding networks are poorly equipped. There is uncertainty about where support can be found, or nobody takes responsibility for support, even though survivors themselves have called for help.

### Embodied suffering that emerged as a memory

The experience of suicide involves discovering one’s vulnerability and fragility. The suffering is shared and involves self-examination and embodied memory. Moreover, suffering can include ambivalence about guilt, long-term pain resulting in feelings of emptiness, insensitive treatment, stigma, and a shadow over life. The experience involves intense sensory and emotional impressions, which unavoidably communicates the presence of death.

Survivors experience ambivalence about guilt, and professionals ruminate on whether something should have been done differently to prevent suicide. This self-examination repeatedly raises questions about whether something could have been done to prevent suicide. The reason why a person died due to suicide remains unclear. Persistent reflections on alternative courses of action and an extended search for meaning often characterise the aftermath, sometimes lasting for years and leaving survivors with enduring uncertainty. Later-emerging intrusive thoughts may further contribute to a fragmented sense of time.

*Brooding, and all of these questions. We’ve all asked the same questions/.../I think it was you who said, “It never goes away.” But I’ve started it; this will never go away. So they say, “Time heals all wounds” and all that silly stuff, but it doesn’t go away. I’ll carry this experience with me until I draw my last breath. That’s the way it is, and somehow you can find a sort of peace in it. My life is now different than it was before, and it will always be so.*

Searching for answers and ways to make sense of the loss gradually contributes to small fragments of increased knowledge and understanding of what has happened. Family members often return to the same questions repeatedly; while some feel or believe that they have found answers, others remain in complete uncertainty. Among the bereaved, some may come to respect or even accept the decision made by the deceased, despite its devastating nature.

*In processing and after, I thought it [his body] got old in a way. It wasn’t any age [in number of years], but he lived, and he tried to live. I think he had been unwell for many years.*

It is difficult to be the messenger of death, and a serious matter to convey an irrevocable announcement that someone has died, knowing that it will affect the lives of others. A death notification is a sudden event for survivors that brings extreme focus and bottomless chaos. The pain is related not only to the fact that someone has died due to suicide, but also to the emptiness that comes afterwards and the feeling of not being important enough to live for. It is difficult to confirm what happened to others, to say the words “death” and “suicide”, and to describe how the person died. There can be a sense of relief over what has happened, and at the same time, great sadness. The pain may include thoughts about not wanting to live anymore. Loss of energy and confusion add suffering, and once in an emotional abyss, it is difficult to take initiative.

*For me, who stood there in that “hole” and looked up and thought, can I ever reach the light again?*

Being among others, even professionals, can evoke fears among survivors of experiencing insensitive conduct, ignorance, and inadequate comprehension of their situation. The stigma around suicide, the taboo of talking about it, feelings of guilt, and pure curiosity, comments, and expectations from others all combine to make survivors silent, lonely, and isolated. Sometimes people are perceived to behave in a way that makes everyone uncomfortable, and as being more interested in the details of the incident than wanting to help. Survivors’ lives have been affected in many ways, changing their approach to life, while professionals have become aware of the transience of life. Suicide is a traumatic, deeply moving, surreal experience that lies far outside the comfort zone of most professionals. The presence of a wounded body, combined with their own emotional reactions and those of survivors and colleagues, leaves lasting impressions etched into memory with a profound intensity. Reality comes painfully close through encounters with life stories that leave embodied traces. These experiences remain not only as memories but also as felt impressions that shape a professional’s understanding of vulnerability, suffering, and the fragile nature of human connections. A response police officer expressed the following:

*Some details remain, usually something that stands out from the event. Maybe you don’t remember it all the time, but when you’re reminded of it, it all comes back. They’re always present somewhere.*

For survivors, the loss and grief remain and affect life forever; in work, privately, and in relation to people around them.

### Strong drive to maintain human dignity

A person is much more than the way they die. The maintenance of human dignity can be seen in death notifications that are experienced as dignified and respectful, as well as through the courage to support, comfort, and relieve suffering. By respecting community and interdependence, one stands up for the human dignity of the deceased, others, and oneself.

The memory of the deceased person can be kept alive by talking about the person and associating them with things in everyday life that act as reminders of them. This is not about denying that the person died by suicide, but instead that there was so much more to them than the way they died, thus emphasising human value above all else. The memory of the deceased is promoted by highlighting the joyful and not only the sad, which can be further broadened by narratives from others.

*Survivor 1: “He was incredibly good at sports, and that’s lived on in them [children]. So he’s still involved in everyday life, and that’s felt very nice.”*

*Survivor 2: “Well, because it’s the living that you must hold on to. Living—because it’s easier, as if suicide and mental state weren’t everything. However, everything else was more. And that’s what helps you.”*

*Survivor 3: “They were a whole human being.”*

There is a will to do the right thing and stand up for human dignity. This manifests in several ways; for example, by delivering a death notification that is dignified and respectful and getting it to the right person as quickly as possible. Humanity is conveyed even through a professional approach to performing formal tasks and taking care of practical things that are considered to help survivors. All this requires courage to stay and encounter survivors in the midst of tragedy, and to be human by being compliant, responsive, and empathetic. A response police officer described his way of encountering survivors:

*Instead, I usually express my condolences. They were allowed to express their grief. I usually think about it when I’m out delivering death notifications, that I usually express myself like this: “I’m sorry for your loss.”*

Survivors need to manage others’ emotions by defending, protecting, and standing up for deceased family members. This sometimes involves answering questions and correcting misunderstandings. In other cases, it is about protecting their own values through having a moment of distance from their thoughts; for example, during everyday routines, work, or meditation.

*For me, it [the work] was salvation. You set the clock, you get up, and then you shower and brush your teeth. Be at work. [Support from colleagues] A colleague said, “Now we’re going to have lunch, you too! All of us.” If I had lain down, I wouldn’t have got up.*

Maintaining human dignity can also involve limiting the opportunity to see the deceased and the place of suicide, while doing this in a respectful and dignified way. This is partly for the survivors, and partly for the protection of the public and professionals who later arrive at the scene. A response police officer provided an example of how human dignity can be maintained:

*I may have sometimes said that it might be appropriate for you to bring in a cleaning company or a remediation company first, before you go in and do what you have to do. Otherwise, trauma occurs within the trauma area.*

Daring to talk about suicide becomes a vindication of a deceased person’s human dignity. Survivors understand each other more comprehensively, and can grasp the extent of what has happened. They also have the courage to ask each other painful and challenging questions. By sharing their thoughts and experiences, they help each other protect human dignity. This unwanted experience means that they dare to talk about death and find it easier to make contact, ask other survivors about their wellbeing, and find courage to be in the existential space.

## Discussion

This study, based on the phenomenon of loss of human life by suicide, illuminates both powerlessness and responsibility, along with an existential dimension, suffering, and just being together as humans. Previous studies have indicated uncertainty in these situations for the professionals involved (De Leo et al., 2022; Foggin et al., 2016; Hofmann et al., 2022; Møller et al., 2023; Smith-Cumberland & Feldman, 2005; Stewart et al., 2000; Tataris et al., 2017). The present study identifies the difficulties of losing a person through suicide—being emotionally unprotected and facing existential challenges—with all that this entails. These findings are in good agreement with (Travelbee, 1971) statement that “every human being suffers because he *is* a human being, and suffering is an intrinsic aspect of the human condition” (*p*. 61). Travelbee defines caring as a process of interpersonal aspects, and in her human-to-human relationship model she describes the nurse and the patient as human beings who relate to each other. In a previous study, ambulance personnel, response police officers, and GPs expressed that this complex situation involved both taking responsibility (or not doing so) and, above all, the capacity to be compassionate and having the courage to meet the ethical demands of survivors (Nilsson et al., 2017). The findings of the present study make it clear that suffering and existential questions evoked compassion in professionals and responsibility for other family members in survivors. Their concern and compassion for each other as humans, highlighted through the depth of suffering and tragedy, were evident.

Existential issues are part of everyday work with vulnerable people, but suicide seems to take hold at a deeper level, causing existential suffering. Survivors and professionals share this suffering. Among professionals, vulnerability manifests as a form of compassion, with pushed boundaries which give them a feeling that “*it could have been me*”. In comparison to pity, where the other is seen as weak, inferior, or “less” in some way, compassion requires a person to transcend their inner boundaries (Wiklund Gustin, 2017). It involves a transition in the professional role, from initially having a clear and strict focus based on norms and ideas about one’s professional role, to going beyond and becoming a compassionate person and a fellow human being in the difficult existential situation that a suicide entails. This means that it is not possible to “just” be a “professional practitioner” in the narrow sense, and that it would be wrong or bad not to show care and be a fellow human being.

Therefore, there is no clear boundary between the professional and fellow human roles in encounters with suffering survivors. Bremer et al. (2012) describe the experiences of emergency medical service personnel caring for families when patients experience cardiac arrest and sudden death. The professionals in these situations need to accept their own vulnerability and the demand for humanity, and find a balance between closeness and distance in encounters with survivors. This agrees with the findings of the present study, as it shows the importance of accepting one’s own vulnerability and humanity. Thorup et al. (2012) described courage as having a significant role in nurses’ ability to engage in situations with suffering people, and stated that nurses’ vulnerability and suffering shape and strengthen their courage. Therefore, in encounters with survivors, it is important to have courage to meet the difficult situation of existential suffering as a vulnerable human being. Being present as a human being may at first appear simple, yet it is a significant task that requires moral courage. Moral courage refers to the willingness to expose one’s own vulnerability to do good for another person who is suffering. Encountering suicide survivors, this dimension becomes particularly evident—the combination of meeting a vulnerable person while simultaneously acknowledging one’s own vulnerability constitutes a fundamental aspect of professional caring. Moral courage is not an innate personal trait; rather, it can be learned, developed, and strengthened over time. Both individual and organisational factors can either promote or inhibit the expression of morally courageous behaviour (Pajakoski et al., 2021).

The powerlessness that arises from feelings of inadequacy and an incomprehensible situation can hinder a person’s will to act. For survivors, feelings arise from being torn between acting and not acting, and shouldering responsibility in the situation while simultaneously bearing the loss. For professionals, encountering suicide brings uncertainty about their ability to shoulder their responsibility. The precarious existential nature of suicide is unfamiliar and may evoke the desire to withdraw from the situation. The discomfort of being confronted with such profound suffering can also trigger recognition—a resonance with one’s own past reflections on life and death that is deeply human.

The powerlessness and insecurity described by professionals can be ameliorated by intervention. In Levinas, (1998) description of encounters between people, he states that the carer’s responsibility for genuine encounters takes place face-to-face with the other. This encounter with the face of the other is a privileged phenomenon, in which the closeness and distance of the other person are both palpable and strong. According to Levinas, the face is naked: exposed, vulnerable, and open to both recognition and harm. This calls for us to exhibit ethical responsibility, even when we do not fully understand or recognise the other. This responsibility includes accepting another person’s radical otherness, which may appear alien or unfamiliar. In encountering what is not me, I am ethically obligated to respond—to take responsibility—even in the absence of mutual recognition. Travelbee, (1971) argued that everyone experiences common human experiences, such as illness and loss. These experiences, although common, are individual and cannot be fully understood by any other individual. According to Levinas, (1998), however, the other’s face helps us understand what we should do, and it is in the encounter with the otherness of others that our own horizon is challenged and moral responsibility for the other is born. This does not mean taking responsibility *from* the other, but taking responsibility *for* the other (Løgstrup, 2007).

A professional needs to exercise responsibility, and as a society we need to take responsibility for survivors receiving initial support in the most vulnerable situation of their lives. Survivors can continue to live, and their inner strength clearly emerges in the findings of the present research. However, time is also of decisive importance. Some of the participating survivors were able to find comfort and strength in encounters with like-minded people; however, at the beginning, when the news of death was fresh, the survivors were in a state of chaos. In such situations, children in the survivor’s family are completely dependent on the ability of others to provide the support they need, and there is a great risk that children will be left alone and without support.

The survivors in the present study were forced to take on the role of messenger of death, conveying the news to loved ones; this was a responsibility with a dual role that also weighed on them. Even for the professionals, being “the messenger of death” was perceived as a burden, as they knew that the living situation of the survivors would forever be changed by the message delivered. (Tranberg & Brodin, 2023) compared breaking bad news to being on stage, not only as a professional but also as a human being.

It is important to remember that in this situation, no one can do it better, but everyone can be the fellow human being that is required. It is in facing another human being, in the space between, that the encounter occurs; here, we can meet each other with care, warmth, and kindness, and understand the other’s needs. In addition, research has shown that patients feel forgotten when they are not treated with compassion (Wiklund Gustin, 2017). In this study, everyone, regardless of their role, had a strong drive to uphold human dignity. Dignity was established through care and encounters with vulnerable people. The professionals also expressed a desire to alleviate suffering, as well as a feeling of being able or willing to do something for the other in this tragedy.

## Methodological considerations

A previous study has merged interpretive phenomenology with community-based participatory research (Bush et al., 2019), but to the best of our knowledge, no studies have combined RLR and PAR into a phenomenological participatory action research approach. The ontology in RLR is drawn from (Husserl, 1970/1936) and (Merleau-Ponty, 2002/1945) lifeworld theory, which advocates the phenomenological approach of “going back to the things themselves” in order to understand the investigated phenomenon. In line with RLR, the researchers used the methodological principles of openness, curiosity, and bridling throughout the process (Dahlberg et al., 2008), with an increased focus on the bottom-up perspective of PAR. Phenomenological PAR was selected because it aligns well with both the study design and subject matter, even though the loss of human life by suicide can be understood as a complex and sensitive phenomenon that is difficult to talk about regardless of methodological design. This approach facilitates close collaboration between participants and researchers, enabling the integration of prior knowledge and fostering an active reflective process aimed at deepening understanding and uncovering meaning. The researchers therefore chose to combine PAR (Reason & Bradbury, 2008) with RLR (Dahlberg et al., 2008), to understand the phenomenon by making use of lived experience and hence existing knowledge. RLR served as the primary guiding approach, while PAR was employed as a methodological tool. Through group dynamic processes and recurring meetings, conditions were created for reflection, the exchange of experiences, and the progressive development of participants’ knowledge and capacity for action. The process was guided by the core principles of RLR, and the researchers adopted a responsive and flexible approach, closely attending to participants’ contributions, experiences, and priorities throughout the recurring group sessions.

PAR was not used in its entirety; rather, the methodological approach was inspired by PAR incorporating a participatory and iterative reflective group process. A key distinction was that participants did not assume the full role of co-researchers. Instead, the responsibility for scientific RLR analysis remained with the researchers. Another part was that in PAR processes, it is customary to challenge participants with theories, conceptual understandings, and/or research findings (Heron & Reason, 2008). In this study, however, we deliberately chose *not* to challenge the participants with predetermined solutions or external research. Instead, the participants were engaged with the rich, touching, and nuanced narratives that emerged, along with the diverse perspectives expressed within the groups. Through these discussions, the participants collectively generated substantial and meaningful content. (Dahlberg, 2006) states that data must be rich in meaning to find the essence of a phenomenon. The combined cycles of the PAR process and the RLR approach revealed deep and rich data. Demographic variation among the participants also contributed important and varied experiences (Dahlberg et al., 2008), and led to increased understanding and a nuanced overall picture of the phenomenon.

The PAR method can be perceived by participants as demanding, which can be a reason for dropout. There are also other natural reasons for dropout, including illnesses such as colds and COVID. This study included only a few doctors, possibly due to the high workload and time pressure involved in their work. Moreover, inclusion was selective: only those who wanted to participate and were interested were included. Consequently, some nuances of the phenomenon may have been missed, but the essential structure of meanings would probably remain the same.

PAR provides opportunities for learning (Reason & Bradbury, 2008), which was evident in both participant groups (survivors and professionals). Between the sessions, some of the professionals encountered other survivors outside the study in their work and had new experiences to share with their colleagues. They also had an increased understanding of their feelings about new situations. Survivors described new lessons learned from the study, which were confirmed by others in similar situations, and they expressed that they no longer felt alone.

One of the researchers (CN) works in the ambulance service and hence may be considered an insider researcher, which could have introduced potential risks such as bias, role conflicts, ethical dilemmas, and reduced objectivity. To mitigate these risks, the last author (EB), who held a more distanced outsider position, was initially involved in the research process to provide critical support and oversight. EB introduced the PAR approach to the participants and helped establish a reflective and ethically sound foundation for the study. After this initial phase, CN continued the phenomenological PAR process. All researchers were familiar with the phenomenological methodology, and all of them contributed reflective input throughout the study. Importantly, they remained aware that the participants’ lived experiences guided the analysis, which helped maintain a participant-centred and ethically grounded approach. EB’s outsider perspective served as a counterbalance to CN’s insider position, which contributed to reflection and guarding against unconsciously biased perspectives. The risk of role conflict was therefore avoided while maintaining the insider researcher’s advantage of familiarity with the context (Coghlan & Shani, 2008). It is important to use self-reflection to critically examine one’s own and others’ experiences (Brydon-Miller, 2008), which has been a natural part of the research process.

There is a lack of studies on the experiences of male survivors (Maple et al., 2014), and so one strength of the present study is that it included both men and women. Another strength is that the survivors had different types of kinship relationships with the deceased, which has also been missing in previous studies (Sveen & Walby, 2008). It can also be considered a strength that the participants were not recruited from existing support groups (Maple et al., 2014). Yet another strength is that the results are based on the perspectives of both survivors and professionals, where similarities are confirmed from different perspectives. However, since the participants comprised only those who had the energy and strength to talk about their experiences and who felt comfortable and safe doing so, some perspectives may have been missed.

Storytelling is a particularly powerful tool when multiple stories about the same topic are presented by different participants. This method values the unique personal experience (Labonte et al., 1999), and creates space for the individual’s lived experience to be disclosed and understood and a bottom-up perspective to be achieved. (Swantz, 2008) states that knowledge acquired through PAR must become part of people’s lives. In our study, knowledge was created as a natural part of the process, which was likely made possible by using phenomenological PAR in combination with RLR, as the researchers actively took a step back to promote the participants’ own voices.

Another strength was that the groups were not mixed, which meant that each group included either professionals or survivors, but not both. This also meant that a group could explore more deeply together, and the survivor and professional groups could ultimately learn from each other. All the participants took responsibility for the process and were deeply engaged. The variation in time elapsed since the suicide was considered a strength because it enabled access to a wide range of lived experiences, thereby enriching the exploration of the phenomenon in line with the principles of RLR (Dahlberg et al., 2008).

## Conclusion

The perspectives of suicide survivors and professionals made it possible to see the depth of meanings in their experiences, highlighting that everyone is vulnerable to and affected by the existential challenges that follow suicide. In such moments, humanity hangs by a thread. Professionals need training to prepare to face suicide with awareness of their own vulnerability and the emotional impact of such events, where a model for reflection can be beneficial for professionals. Survivors in turn need professionals who have the moral courage to encounter them not only in their role, but also as fellow human beings. Furthermore, acknowledging that the survivor is a person who may need support over time, became clear with the realisation that grief and existential suffering do not follow a fixed timeline.

## Data Availability

The data supporting the findings of this study are not publicly available due to ethical and legal restrictions. The dataset contains personal and sensitive personal data collected from human participants and is therefore subject to the Swedish Ethical Review Act (SFS 2003:460) and the EU General Data Protection Regulation (GDPR; Regulation (EU) 2016/679). Under these regulations, public data sharing is not permitted where it could compromise participant privacy or violate the conditions of ethical approval. Even in anonymised form, there is a risk of re identification. Aggregated data supporting the conclusions are included within the article. Access to controlled data may be considered on reasonable request, subject to ethical review and applicable legal requirements.
